# Harnableitung beim alten Patienten (80+)

**DOI:** 10.1007/s00120-024-02384-6

**Published:** 2024-07-16

**Authors:** D. Oswald, T. R. W. Herrmann, C. Netsch, B. Becker, G. Hatiboglu, R. Homberg, J. T. Klein, K. Lehrich, A. Miernik, P. Olbert, D. S. Schöb, K. D. Sievert, J. Herrmann, A. J. Gross, M. Pallauf, S. Deininger, C. Ramesmayer, J. Peters, L. Lusuardi

**Affiliations:** 1 Universitätsklink für Urologie und Andrologie, Salzburger Landeskliniken, Paracelsus Medizinische Universität Salzburg, Müllner Hauptstraße 48, 5020 Salzburg, Österreich; 2 Urologie, Abteilung für Urologie, Kantonsspital Frauenfeld, Frauenfeld, Schweiz; 3 Klinik für Urologie, Universitätsklinikum Schleswig-Holstein, Campus Lübeck, Ratzeburger Allee 160, 23538 Lübeck, Deutschland; 4 Asklepios Klinik, Barmbek, Hamburg, Deutschland; 5 Heilbronn, Deutschland; 6 Klinik für Urologie, Kinderurologie und Uro-Gynäkologie, St. Barbara-Klinik Hamm-Heessen, Hamm, Deutschland; 7 Urologie, Kantonsspital Münsterlingen, Münsterlingen, Schweiz; 8 Abteilung für Urologie, Uniklinikum Ulm, Abteilung für Urologie und Kinderurologie, Ulm, Deutschland; 9 Klinik für Urologie, Vivantes Auguste-Viktoria-Klinikum, Berlin, Deutschland; 10 Medizinische Fakultät, Klinik für Urologie, Universitätsklinikum Freiburg, Freiburg, Deutschland; 11 BRIXSANA private clinic, Brixen, Italien; 12 UKOWL, Campus Klinikum Lippe, Detmold, Deutschland; 13 Klinik für Urologie und Urochirurgie, Universitätsklinkum Mannheim, Medizinische Fakultät Mannheim, Universität Heidelberg, Mannheim, Deutschland

**Keywords:** Muskelinvasives Blasenkarzinom, Geriatrischer Patient, Zystektomie, Ileum-Conduit, Ureterokutaneostomie, Muscle-invasive bladder cancer, Geriatric patient, Cystectomy, Ileal conduit, Ureterostomy

## Abstract

Bei steigender Lebenserwartung gibt es zunehmend ältere (≥ 80 Jahre) PatientInnen mit der Diagnose eines muskelinvasiven Blasenkarzinoms. Therapie der Wahl ist die radikale Zystektomie mit Harnableitung (mit neoadjuvanter Chemotherapie, sofern belastbar). Die Auswahl der richtigen Harnableitung in Abwägung von Morbidität gegenüber Funktionalität und Lebensqualität stellt eine Herausforderung dar. Das kalendarische Alter allein ist nicht entscheidend. Wegweisend ist v. a. eine adäquate präoperative Begutachtung mit Blick auf medizinische Besonderheiten sowie physische und kognitive Einschränkungen. Standardmäßig wird bei älteren PatientInnen das Ileum-Conduit als inkontinente Harnableitung eingesetzt, da der Eingriff eine geringere Komplexität und Operationsdauer als eine kontinente Harnableitung aufweist. Fitte PatientInnen mit adäquater Lebenserwartung und ausreichender Compliance können jedoch auch im hohen Alter Kandidaten für kontinente Harnableitungen sein. Die Ureterokutaneostomie mit Harnleiterschienendauerversorgung ist eine wichtige Alternative für multimorbide PatientInnen mit hohem perioperativem Risiko. Wichtig ist v. a. eine gute präoperative Aufklärung, sodass PatientInnen eine informierte Entscheidung treffen können.

## Abstract (en)

With increasing life expectancy there is also an increased need for the management of older (≥ 80 years) patients with the diagnosis of muscle-invasive bladder cancer. Radical cystectomy with urinary diversion is the state of the art treatment (with neoadjuvant chemotherapy, as long as the patient is fit enough). Choosing the best urinary diversion with respect to morbidity compared to functionality and quality of life remains a challenge in these patients. Physical age alone is not decisive for making a decision. A thorough preoperative assessment of medical features, physical and cognitive impairments is more important. Older patients are generally provided with an ileal conduit as an incontinent urinary diversion, as the intervention involves reduced operating times and complexity compared to continent urinary diversions; however, in the case of good health status with an adequate life expectancy and sufficient compliance, continent diversions may be considered even in aged candidates. In the case of multimorbid patients with a high perioperative risk, ureterostomy with permanent ureteric stents is an important alternative. Most importantly, a thorough preoperative counselling enables patients to reach an informed decision.

## Lernziele

Nach Absolvieren dieser Fortbildungseinheit …

- können Sie epidemiologische Daten zum Auftreten des Harnblasenkarzinoms bei älteren Patienten benennen.
- kennen Sie Definitionsweisen und Screeningmethoden des älteren bzw. komorbiden Patienten und deren Schwierigkeiten im Hinblick auf die Studienlage zur Zystektomie.
- können Sie Indikationen und Kontraindikationen für spezifische Harnableitungen beschreiben.
- können Sie Vor- und Nachteile unterschiedlicher Harnableitungen beim älteren Patienten im Hinblick auf perioperative Risiken gegeneinander abwägen.
- kennen Sie Vor- und Nachteile unterschiedlicher Harnableitungen beim älteren Patienten im Hinblick auf Funktionalität und Lebensqualität.

## Einleitung

Das Blasenkarzinom zählt zu den häufigsten Krebserkrankungen weltweit und nimmt insbesondere in Gesellschaften der industrialisierten Welt mit höherer Alterserwartung eine zunehmende Rolle ein. Dies ist dem relativ hohen Alter bei Erstdiagnose und der **demographischen Entwicklung**

Demographische Entwicklung,

mit stetiger Zunahme der älteren Bevölkerungsschicht, zuzuschreiben. Im muskelinvasiven Stadium ist die radikale Zystektomie mit Harnableitung nach neoadjuvanter Chemotherapie der therapeutische Goldstandard. Dieser Eingriff hat eine hohe perioperative Morbidität und Mortalität, wobei der Art der Harnableitung hierbei eine entscheidende Rolle zukommt. Die Art der Harnableitung und ihre möglichen Komplikationen bestimmen aber auch wesentlich die Lebensqualität der PatientInnen. Besonders bei älteren Personen scheint hier oft ein Kompromiss nötig.

## Epidemiologie

**Abb. 1:**
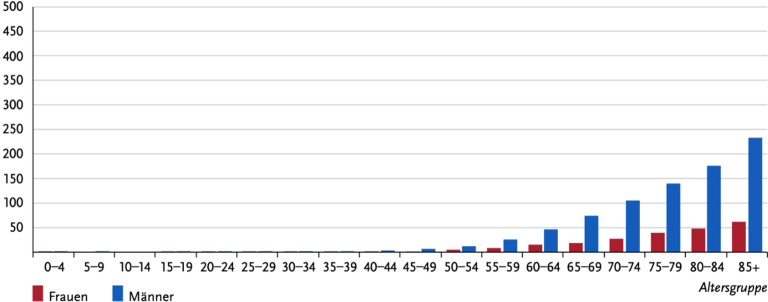
Altersverteilung bei Erstdiagnose eines Blasenkarzinoms in Deutschland 2019/2020 gemäß Daten des Robert-Koch-Instituts (RKI). (Nach [2])

Das Harnblasenkarzinom ist weltweit der neunthäufigst diagnostizierte Tumor mit einer geschlechterspezifischen Verteilung von 3:1 für Männer und Frauen. Im deutschsprachigen Raum liegt das Harnblasenkarzinom sogar an fünfter Stelle. Die altersstandardisierte Inzidenzrate im deutschsprachigen Raum wird von der World Health Organization (WHO) mit 12,2 angegeben [1]. Das Alter bei Erstdiagnose betrug in Deutschland gemäß dem Bericht des Robert-Koch-Instituts 2019/2020 74 Jahre. Bei über 80-Jährigen steigt die Inzidenz im Unterschied zu jüngeren PatientInnen rasant an (Abb. 1; [2]). 25 % der erstdiagnostizierten Blasentumoren sind hier bereits muskelinvasiv [3].

## Zystektomie und Harnableitung

Therapie der Wahl des muskelinvasiven Blasenkarzinoms ist die **radikale Zystektomie**

Radikale Zystektomie

mit pelviner Lymphadenektomie und **Harnableitung**. Cisplatinfitte PatientInnen sollen vorher eine neoadjuvante Chemotherapie erhalten. Dies gilt grundsätzlich auch für ältere PatientInnen, stellt jedoch ein eigenes Thema dar, das in diesem Artikel nicht diskutiert werden soll. Als blasenerhaltende Alternative besteht die Option der trimodalen Therapie mit möglichst radikaler TUR(transurethrale Resektion)-Blase

TUR-Blase

und konkomitanter Radiochemotherapie. Diese erreicht aber nach wie vor nur in einem selektionierten Patientengut vertretbare onkologische Ergebnisse. Geeignet sind v. a. PatientInnen mit niedrigen Tumorstadien ohne zusätzliche Risikofaktoren (multifokale Tumoren, Involvierung der Ostien bzw. Hydronephrose, konkomitantes Carcinoma in situ, geringes Blasenvolumen; [4]).

Älteren PatientInnen wird die radikale Operation aus Sorge vor Komplikationen oft vorenthalten. In einer Datenbankanalyse von PatientInnen über 80 Jahren mit muskelinvasivem Blasentumor lag der Anteil derer, die zystektomiert wurden, bei nur 7 % [5]. Auch diese Gruppe profitiert jedoch von einer radikalen Operation im Sinne eines erhöhten tumorspezifischen Überlebens [6]. Als Faustregel wird in der Literatur eine Lebenserwartung von 2 Jahren als Voraussetzung für eine kurative Therapie des muskelinvasiven Blasentumors vorgeschlagen [7].

Die Zystektomie gilt als **komplikationsträchtige Operation**. In einer retrospektiven Analyse mehrerer europäischer Zentren wurden eine 30- und eine 90-Tages-Mortalität von 2,7 bzw. 9 % berichtet [8]. Zahlen zur Komplikationsrate variieren stark je nach Serie, wobei v. a. Langzeitfolgen oft auf die Art der Harnableitung zurückzuführen sind [9]. In einer aktuellen systematischen Übersichtsarbeit hatten PatientInnen über 80 Jahre, verglichen mit jüngeren PatientInnen, eine deutlich erhöhte perioperative Mortalität mit einer Odds Ratio (OR) von 2,82 und 3,34 für 30 bzw. 90 Tage postoperativ. Die perioperative Komplikationsrate war mit einer OR von 1,2 ebenso leicht erhöht [10].

Die Harnableitung kann in kontinent und inkontinent sowie orthotop und heterotop eingeteilt werden. Die häufigsten Harnableitungen sind das **Ileum-Conduit**

Ileum-Conduit

(IC; heterotop und inkontinent) und die **Neoblase**

Neoblase

(NB; orthotop und kontinent). In einzelnen Zentren werden auch **katheterisierbare Pouches**

Katheterisierbare Pouches

(heterotop und kontinent) angeboten. Das meistverwendete Darmsegment ist das Ileum, aber auch das Kolon oder die Appendix vermiformis können für die Harnableitung verwendet werden. Die **Ureterokutaneostomie**

Ureterokutaneostomie

(UC; heterotop und inkontinent) stellt die einfachste dauerhafte Option der Harnableitung dar, da kein Darm zur Rekonstruktion verwendet wird. Im Regelfall muss mit einer **Harnleiterschienendauerversorgung**

Harnleiterschienendauerversorgung

gerechnet werden [4].

## Der alte Patient

Wer ist alt? Eine exakte Definition für den älteren Patienten zu treffen, ist schwierig. Durch medizinischen Fortschritt und verbesserten Lebensstandard ist die Alterserwartung stetig gestiegen [11]. Für die Abschätzung perioperativer Risiken der Zystektomie ist aber ohnehin nicht allein das kalendarische Alter, sondern v. a. das Vorliegen geistiger und körperlicher Einschränkungen relevant [12].

Die Deutsche Gesellschaft für Geriatrie (DGG), die Deutsche Gesellschaft für Gerontologie und Geriatrie (DGGG) und die Bundesarbeitsgemeinschaft Geriatrischer Einrichtungen (BAG) teilen eine gemeinsame Definition des geriatrischen Patienten (Tab. 1; [13]).

**Tab. 1:** Definition des geriatrischen Patienten

Definition des geriatrischen Patienten
*Entweder*
Geriatrietypische Multimorbidität (vorrangig vor kalendarischem Alter)
Höheres Lebensalter (überwiegend ≥ 70 Jahre)
*Oder*
≥ 80 Jahre
Alterstypische erhöhte Vulnerabilität (Frailty)

Man stellt fest, dass das kalendarische Alter erst ab dem Alter von 80 Jahren (sog. „oldest old“) als alleiniges Kriterium zur Definition herangezogen wird. Wichtiger sind Multimorbidität, Polypharmazie sowie die sog. **Frailty**

Frailty,

die im Deutschen mit dem Begriff „Gebrechlichkeit“ übersetzt werden könnte.

Gebrechlichkeit meint eine altersspezifisch erhöhte Vulnerabilität gegenüber externen Stressoren und kann zu einer maßgeblichen Einschränkung der eigenständigen Ausübung täglicher Aktivitäten führen. Physische Zeichen der Gebrechlichkeit sind eine verlangsamte Gangart, körperliche Schwäche, physische und psychische Erschöpfung sowie verminderte körperliche Aktivität [14]. Der Zusammenhang der Gebrechlichkeit mit postoperativer Morbidität und Mortalität ist in der Literatur gut dokumentiert, wobei unterschiedliche Instrumente zur Messung vorliegen [15]. Die europäische Leitlinie zum Management des muskelinvasiven Blasenkarzinoms empfiehlt zum Beispiel den **G8 Questionnaire**

G8 Questionnaire

oder die **Clinical Frailty Scale**

Clinical Frailty Scale

(CFS) zur präoperativen Evaluierung [4].

Auch das Vorliegen relevanter Komorbiditäten stellt ein altersunabhängiges, erhöhtes perioperatives Komplikationsrisiko dar [16]. Der altersadaptierte **Charlson Comorbidity Index**

Charlson Comorbidity Index

(CCI) ist das am weitesten verbreitete validierte Screeningtool, das auch in der präoperativen Abklärung der Zystektomie eingesetzt werden sollte [4].

Zur Evaluierung einer kognitiven Einschränkung empfiehlt die EAU(European Association of Urology)-Leitlinie den **Mini-COG-Test**

Mini-COG-Test,

der mit einer Dauer von etwa 5 min alltagstauglich durchgeführt werden kann [4].

Bei Auffälligkeiten im Rahmen des präoperativen Screenings kann ein umfassendes **geriatrisches Assessment**

Geriatrisches Assessment

zur präoperativen Einschätzung und Optimierung durch KollegInnen der Geriatrie durchgeführt werden.

Eine direkte Empfehlung zur Art der Harnableitung ist aus keinem dieser Tools abzuleiten [4].

Geriatrische PatientInnen sind aufgrund der genannten Faktoren durch eine reduzierte Reservekapazität in allen Organsystemen gekennzeichnet. Diese Einschränkungen müssen im Hinblick auf therapeutische Konzepte berücksichtigt werden. Das kalendarische Alter allein ist zwar wenig aussagekräftig, mit steigendem Alter steigt jedoch auch das Risiko für Gebrechlichkeit und Komorbidität.

Im Hinblick auf die Zystektomie ist es schwierig, eine allgemeingültige Altersgrenze für den „alten Patienten“ zu finden. Viele Studien verwenden unterschiedliche Grenzwerte von 60 bis 85 Jahren [17]. In diesem Artikel soll es, auch in Anlehnung an die Definition des geriatrischen Patienten, vorrangig um PatientInnen ab 80 Jahre („oldest old“) gehen.

## Frequenz unterschiedlicher Harnableitungen

In den meisten Fallserien älterer PatientInnen, die zystektomiert wurden, zeigte sich eine deutliche Präferenz der **inkontinenten Harnableitung**

Inkontinente Harnableitung.

Froehner et al. zeigten in einer Übersichtsarbeit zu Harnableitungen beim älteren Patienten ein Verhältnis von 30 zu 70 % für die **kontinente**

Kontinente Harnableitung

bzw. die inkontinente Harnableitung. Analysiert wurden in dieser Studie allerdings verhältnismäßig junge PatientInnen, was den vergleichsweise hohen Anteil an kontinenten Harnableitungen erklärt. Die meisten Studien schlossen PatientInnen ab 70 Jahren ein; in 2 Serien lag die Definitionsgrenze des alten Patienten überhaupt bei nur 65 Jahren [17]. PatientInnen, die eine kontinente Harnableitung erhielten, waren jünger als PatientInnen mit inkontinenter Harnableitung [18, 19].

Mehrere retrospektive Serien von PatientInnen über 80 Jahre, welche zystektomiert wurden, sind vorhanden, teilweise mit jüngeren Vergleichskohorten. Das IC ist die meistgenutzte Harnableitung. Die UC nimmt einen höheren Stellenwert ein als bei jüngeren PatientInnen, während die NB nur vereinzelt in Zentren durchgeführt wird [12, 20, 21, 22, 23]. Die Daten zu katheterisierbaren Pouches bei mindestens 80-jährigen PatientInnen beschränken sich auf wenige PatientInnen in älteren Serien [12].

Vergleichende Daten zur Harnableitung von über 80-jährigen PatientInnen fehlen, insbesondere in Bezug auf Komplikationen. PatientInnenserien, die den „älteren Patienten“ etwas weiter fassen, berichten im Allgemeinen keine Unterschiede in der perioperativen Komplikationsrate unterschiedlicher Harnableitungen [18, 19]. Clark et al. beschreiben gering- und höhergradige harnableitungsspezifische Komplikationen (Grad < 3 bzw. ≥ 3 gemäß Clavien-Dindo) in 10 % bzw. 8 % der Fälle. Für PatientInnen ab 70 Jahren konnte gezeigt werden, dass keine Unterschiede zwischen den verschiedenen Ableitungsarten (NB, Pouch, IC) in Bezug auf perioperative Komplikationen vorlagen [12]. Yamanaka et al. berichten von identischen Früh- und Spätkomplikationsraten bei über 80-jährigen PatientInnen im Vergleich zur jüngeren Kontrollgruppe, insbesondere bei harnableitungsspezifischen Komplikationen [23]. Aufgrund des retrospektiven Charakters der Studien repräsentieren diese Zahlen v. a. eine adäquate PatientInnenselektion für die Art der Harnableitung.

## Harnableitungsspezifische Komplikationen

Eine kürzere Operations- bzw. Anästhesiedauer ist allgemein als begünstigender Faktor für einen komplikationsärmeren perioperativen Verlauf akzeptiert. Die Operationszeit einer kontinenten Harnableitung ist in den meisten Serien gegenüber einer inkontinenten Harnableitung signifikant erhöht [24]. Bei der inkontinenten Harnableitungen dauerte die Anlage eines IC in den meisten verfügbaren Kohorten signifikant länger als die UC (im Mittel 80 min). Außerdem bedingte sie auch einen tendenziell längeren Krankenhausaufenthalt [25].

Die UC hat die geringste perioperative Komplikationsrate, verglichen mit anderen Harnableitungen. Im Vergleich zwischen UC und IC wird das Auftreten von Komplikationen mit Clavien-Dindo-Grad 3 oder höher in 27,3 % gegenüber 40 % der Fälle beschrieben. Insbesondere **darmspezifische Komplikationen**

Darmspezifische Komplikationen

wie der postoperative Ileus und die Darmanastomoseninsuffizienz können durch eine UC vermieden werden. Weitere begünstigende Faktoren sind geringerer Blutverlust, geringere Transfusionsrate, kürzere Krankenhausliegedauer und kürzerer Intensivstationsaufenthalt [25].

Die größte Problematik der UC stellt die hohe Inzidenz an **Implantationsstenosen**

Implantationsstenosen

an der Haut dar, weshalb in der Regel eine **dauerhafte Harnleiterschienung** mit regelmäßigen Wechseln etabliert wird. Eine Schienendauerversorgung stellt bekanntermaßen ein höheres Risiko für Niereninsuffizienz, Pyelonephritiden und Bildung von Urolithiasis dar [26].

Eine mögliche Lösung für dieses Problem kann mit einer Omentumummantelung erreicht werden. Sollten die intraoperativen Voraussetzungen passen und ein singuläres Stoma angestrebt werden, wird unter Verwendung von Omentum majus

Omentum majus

der Durchtritt beider Ureteren durch die Bauchdecke allseitig gegenüber der Umgebung abgeschirmt. Das Stoma wird bei diesem Verfahren mit Omentum majus unterfüttert. Diese Technik sorgt zum einen für eine mechanische Grenze der fragilen Ureteren gegenüber dem umliegenden Gewebe und zum anderen für einen ausreichenden nutritiven Nutzen in Bezug auf die Blutversorgung. Beides könnte daher helfen, das Risiko einer Stomastenose

Stomastenose

zu reduzieren und den Verzicht auf eine dauerhafte Harnleiterschienung zu ermöglichen. Für diese und ähnliche Techniken fehlen jedoch belastbare Daten, ob langfristig wirklich auf eine Schienendauerversorgung verzichtet werden kann [27].

Ein weiteres Problem besteht darin, dass nicht immer beide Ureteren lang genug sind, um gemeinsam in einem Stoma ausgeleitet zu werden (einzeln oder als Transureteroureterostomie

Transureteroureterostomie).

Dies kann insbesondere bei adipösen PatientInnen ein Problem darstellen. In solchen Ausnahmefällen müssten 2 Stomata angelegt werden, was mit einer entsprechenden Einschränkung der Lebensqualität einhergeht.

Probleme der orthotopen NB betreffen unter anderem die Funktionalität. Die effektive NB-Entleerung bedingt die bewusste Relaxierung des Sphinkters bei gleichzeitiger Bauchpresse. Die Funktionalität der NB im Sinne einer adäquaten Kapazität, Entleerung und Kontinenz ist nicht unmittelbar postoperativ gegeben, sondern muss über Monate nach dem stationären Aufenthalt hinaus trainiert werden. Kontinenzraten bei orthotoper Harnableitung sind bei älteren PatientInnen niedriger, wobei die berichteten Zahlen in Abhängigkeit von Zentrum und Definition der Kontinenz stark differieren [17]. Eine Studie konnte ein inverses Verhältnis von Alter und Rehabilitationsfähigkeit der Kontinenz nach NB-Anlage zeigen [28]. Bei Hyperkontinenz kann die Einschulung auf **Selbstkatheterismus**

Selbstkatheterismus

erfolgen, um eine Dauerkatheterversorgung zu vermeiden [18, 19]. Eine nervschonende Operationstechnik

Nervschonende Operationstechnik

begünstigt sowohl die Kontinenzraten als auch die Erektionsfähigkeit [28]. Sowohl NB-Entleerung als auch Selbstkatheterismus benötigen eine kognitive und physische Grundfitness, um zielführend umgesetzt zu werden.

Es erscheint logisch, dass die unterschiedlichen Arten der Harnableitung auch unterschiedliche Vorteile bezüglich Kontinenz, sexueller Funktion und Körperbild haben. In Bezug auf die Lebensqualität zeigte sich in einer Studie allerdings kein Unterschied nach 1 Jahr [29].

## Roboterchirurgie

Ziel der **robotischen Zystektomie**

Robotische Zystektomie

ist es, die Morbidität des Patienten zu verringern. Geringere Transfusionsraten und eine geringere Krankenhausaufenthaltsdauer sind beschrieben, allerdings auf Kosten einer deutlich erhöhten Operationszeit, weshalb die Technik bei älteren und vorerkrankten Personen vergleichsweise selten zum Einsatz kommt [30].

Eine retrospektive multizentrische europäische Studie analysierte 164 über 80-jährige PatientInnen im Vergleich zu 1726 jüngeren PatientInnen, die eine robotische Zystektomie mit intrakorporaler Harnableitung erhielten. Es zeigten sich tolerable perioperative Komplikationsraten (Clavien-Dindo-Grad ≥ 3) von 11 und 13 % nach 30 bzw. 90 Tagen, die geringer ausfielen als in der jüngeren PatientInnenenkohorte [20].

Daten zur Harnableitung in diesem Patientengut sind kaum vorhanden; insgesamt scheint aber auch bei robotischen Operationen das IC präferiert zu werden.

## Welche Harnableitung passt für wen?

Es gibt keine validierten Kriterien für die Auswahl der korrekten Harnableitung, da die klinische PatientInnenselektion eine Randomisierung unmöglich macht. Das Ziel muss ein gutes onkologisches und funktionelles Ergebnis unter Erhalt der Lebensqualität sein. Eine retrospektive Datenbankanalyse gematchter PatientInnen zeigte keinen Einfluss der Harnableitung (NB, IC) auf das Gesamt- bzw. das tumorspezifische Überleben [31].

Die Auswahl der Harnableitung wird in den meisten Studien nicht nach vordefinierten Kriterien, sondern gemäß der Expertise des Operateurs getroffen. Wichtig ist die informierte Entscheidung gemeinsam mit dem Patienten. In einer Studie, die ein Bedauern der PatientInnen bezüglich der gewählten Harnableitung (IC oder NB) 6 bzw. 18 Monate postoperativ abfragte, konnte kein Unterschied gezeigt werden. Unabhängig von der Art der Harnableitung waren jedoch diejenigen PatientInnen unzufriedener, die subjektiv unzureichende präoperative Informationen erhalten hatten [32]. Eine wesentliche Rolle spielen Erwartungshaltung und Bedürfnisse des Patienten: Manche PatientInnen wollen aktiv in den Entscheidungsprozess eingebunden werden, andere verlassen sich ganz auf die ärztliche Empfehlung. In eine Fragebogenstudie unter 180 ZystektomiepatientInnen hatten die meisten eine klare Präferenz der Harnableitung. Selbstbewusstere PatientInnen tendierten eher zur kontinenten Harnableitung. Die Mehrzahl (62 %) gab trotzdem an, die Wahl der Harnableitung ganz den betreuenden Ärzten überlassen zu wollen [33].

Im Allgemeinen gilt das IC als Standardharnableitung für ältere PatientInnen, da es eine kürzere Operationsdauer und -komplexität als der orthotope Blasenersatz bedingt [17, 34]. Einige Studien zeigen aber, dass eine NB oder ein katheterisierbarer Pouch auch beim gut selektierten 80-jährigen und älteren Patienten möglich sind [12, 35].

Für PatientInnen mit hohem Narkoserisiko, palliativer Operationsindikation mit eingeschränkter Lebenserwartung, Einzelniere oder Kontraindikationen zur Verwendung von Darm (Verwachsungsbauch, Bestrahlung, entzündliche Darmerkrankungen) stellt die UC mit dauerhafter Harnleiterschienenversorgung eine gute Alternative dar. Bei diesem Patientengut ist auch nicht zwingend von einer Einschränkung der Lebensqualität durch die Harnableitung auszugehen [36].

Die allgemeinen **Kontraindikationen**

Kontraindikationen

für eine kontinente Harnableitung gelten auch beim alten bzw. geriatrischen Patienten (siehe Tab. 2). Besondere Beachtung bei älteren PatientInnen sollte die **Nierenfunktion**

Nierenfunktion

finden, da diese ab dem 40. Lebensjahr kontinuierlich abnimmt [37]. Eine schlechtere Nierenfunktion bedeutet im Umkehrschluss weniger Kapazität, eine metabolische Azidose auszugleichen, die durch Rückresorption von verwendeten Darmanteilen entsteht. Zudem kann sie einschränkend für eine adjuvante cisplatinhaltige Chemotherapie sein.

**Tab. 2:** Absolute und relative Kontraindikationen der kontinenten Harnableitung. (Adaptiert nach [38])

**Absolute Kontraindikation**
Schwere Niereninsuffizienz (GFR < 40 ml/min)
Schwere Leberinsuffizienz
Urethrektomie (bei positivem urethralen Schnellschnitt bzw. Harnröhrenbefall) – NB
Geistige oder körperliche Unfähigkeit zum Selbstkatheterismus
Unmotivierter Patient
**Relative Kontraindikationen**
Darmerkrankungen (Tumor, Kurzdarmsyndrom, M. Crohn)
Stressinkontinenz, geschädigter Rhabdosphinkter
Z. n. pelviner Radiotherapie (z. B. bei Prostata- oder Zervixkarzinom)
Harnröhrenstrikturen

*GFR* glomeruläre Filtrationsrate, *NB* Neoblase

Als relative Kontraindikationen für eine kontinente Harnableitung werden in der Literatur zusätzlich auch ein schlechter Performance-Status, schwere Komorbiditäten und lokal fortgeschrittene Tumorstadien genannt [31, 39]. Auch ein höheres Alter bzw. eine limitierte Lebenserwartung zählen als relative Kontraindikationen, sind jedoch meist nicht genauer definiert [4, 38]. Auch wenn für den individuellen Patienten das kalendarische Alter allein nicht entscheidend sein sollte, scheint ein Alter von 80 Jahren in der Literatur ein guter Grenzwert zu sein, ab dem mit höherer Morbidität bei kontinenter Harnableitung zu rechnen ist. In diesem Kollektiv steigt nach Zystektomie die postoperative 30-Tages-Morbidität und -Mortalität im Vergleich zu jüngeren PatientInnen deutlich [10]. Allerdings ist auch zu erwähnen, dass die Fähigkeit ein Urostoma eigenständig zu versorgen, ebenso mit dem 80. Lebensjahr abnimmt [40].

## Fazit für die Praxis

- Das präoperative Assessment im Hinblick auf Tumorcharakteristika, Vorerkrankungen, psychische und physische Einschränkungen, soziales Umfeld und Patientenwunsch ist unerlässlich, um im ausführlichen Aufklärungsgespräch gemeinsam mit dem älteren Patienten die richtige Harnableitung zu wählen. Hierbei können standardisierte geriatrische Assessments helfen, ihr genauer Stellenwert ist jedoch unklar und sollte Inhalt zukünftiger klinischer Studien sein.
- Die standardmäßige Harnableitung des älteren Patienten ist das Ileum-Conduit, das in den Studien ein akzeptables Nebenwirkungsprofil und eine gute Lebensqualität zeigt. Das kalendarische Alter schließt eine kontinente Harnableitung jedoch nicht gänzlich aus, wobei die Indikation ab einem Alter von 80 Jahren zurückhaltend gestellt werden sollte. Neben den gängigen (relativen) Kontraindikationen für eine kontinente Harnableitung muss besonders auf das Vorliegen einer kognitiven Einschränkung (z. B. Demenz), die physische Fähigkeit zum Selbstkatheterismus, vorbestehende Miktionsprobleme (z. B. Stressinkontinenz) und die Nierenfunktion geachtet werden. Katheterisierbare Pouches sind in einzelnen Zentren auch bei älteren Patienten beschrieben, bleiben aber eine individuelle Lösung.
- Die Ureterokutaneostomie sollte aufgrund der regelhaft benötigten Harnleiterschienendauerversorgung nicht standardmäßig angeboten werden. Sie kann jedoch bei PatientInnen mit palliativer Indikation zur Zystektomie und geringer Lebenserwartung, Kontraindikation zur Verwendung von Darmsegmenten oder hohem perioperativen Risiko eine valide Option zur Reduktion der Morbidität sein. Bei diesen PatientInnen kann bei minimiertem operativen Risiko eine gute Lebensqualität erreicht werden.
- Die Zystektomie mit Harnableitung kann auch bei über 80-jährigen Patienten sicher robotisch durchgeführt werden.

## CME-Fragebogen

Was ist das mediane Alter bei Erstdiagnose eines Blasenkarzinoms in Deutschland?

50 Jahre

61 Jahre

69 Jahre

70 Jahre

74 Jahre

Welche epidemiologischen Charakteristika unterscheiden PatientInnen ≥ 80 Jahre mit muskelinvasivem Blasenkarzinom von jüngeren PatientInnen?

Der prozentuale Anteil der PatientInnen, die eine Zystektomie erhalten, ist gleich.

PatientInnen > 80 Jahre profitieren nicht mehr von einem kurativen Therapieansatz.

Eine Lebenserwartung von 10 Jahren gilt als allgemeine Voraussetzung für einen kurativen Therapieansatz bei PatientInnen ≥ 80 Jahre.

Die perioperative Mortalitätsrate nach Zystektomie ist erhöht.

Die perioperative Morbiditätsrate nach Zystektomie ist niedriger.

Wie kann der Begriff „Frailty“ am besten definiert werden?

Gemeint ist das Vorliegen multipler Vorerkrankungen mit Polypharmazie.

Gemeint ist ein altersbedingter Zustand reduzierter Reserven, der eine höhere Vulnerabilität z. B. gegenüber Operationen bedingt.

Gemeint ist die altersbedingte Gebrechlichkeit, die proportional mit zunehmendem kalendarischen Alter ansteigt.

Gemeint ist ein Zustand reduzierter körperlicher Fitness, bei dem PatientInnen mindestens 50 % des Tages sitzend oder liegend verbringen.

Gemeint ist ein altersunabhängiger Zustand vorübergehend eingeschränkter Funktionalität, z. B. in der Rekonvaleszenzphase nach einer Operation.

Ein 82-jähriger Patient mit auswärts erstdiagnostiziertem Blasentumor kommt zur Therapieplanung in Ihre Sprechstunde. Die medizinische Vorgeschichte scheint ohne wesentliche Vorerkrankungen oder -operationen zu sein. Sie sind sich jedoch nicht ganz sicher, ob der Patient alles versteht, was Sie erklären. Was ist ein simpler Test, den sie selbst für eine erste Abschätzung des kognitiven Status durchführen können?

Charlson Comorbidity Index (CCI)

G8 Questionnaire

Mini-COG

Clinical Frailty Scale (CFS)

Geriatrisches Assessment

Sie schreiben eine CME(„continuing medical education“)-zertifizierte Fortbildung für *Die Urologie* über die Harnableitung beim ≥ 80-Jährigen Patienten. Ihnen fällt auf, dass es kaum vergleichenden Studien zu harnableitungsspezifischen Komplikationen gibt. Immerhin gibt es einige Fallkohorten zur Zystektomie beim 80-jährigen Patienten. Was ist die am häufigsten verwendete Art der Harnableitung in diesem Patientengut?

Neoblase

Katheterisierbarer Pouch

Ileum-Conduit

Ureterokutaneostomie

Ureterosigmoideostomie

Ein 87-jähriger Patient mit muskelinvasivem Blasentumor aus Ihrer Sprechstunde hat sich nach ausführlicher Beratung zur radikalen Zystektomie entschieden. Aufgrund seines Alters, mehrerer abdominaler Voroperationen und einer präterminalen Niereninsuffizienz haben Sie sich gemeinsam für eine Ureterokutaneostomie entschieden. Was ist ein Vorteil dieser Harnableitung?

Durch die direkte Fixierung des Harnleiters mit der Haut ist regelhaft ein direkter Harnabfluss ohne Harnleiterschienung möglich.

Die Operationszeit ist deutlich kürzer als bei einer kontinenten Harnableitung, aber gleich lang wie beim Ileum-Conduit.

Da kein Darmsegment verwendet wird, lassen sich postoperative Komplikationen wie Ileus und Darmanastomoseninsuffizienz besser vermeiden.

Durch Ausleitung der Harnleiter jeweils auf beiden Seiten lässt sich eine bessere Lebensqualität durch einfachere Versorgung erreichen.

PatientInnen mit Ureterokutaneostomie haben eine geringere perioperative Mortalität, benötigen jedoch vermehrt Bluttransfusionen und liegen länger auf der Intensivstation.

Ein 81-jähriger, fitter Patient mit muskelinvasivem Blasentumor kommt zur Zweitmeinung in Ihre Sprechstunde. In der Klinik, in der der Tumor erstdiagnostiziert worden war, wurde ihm nur ein Ileum-Conduit angeboten. Er möchte jedoch eine Neoblase. Was muss der Patient auch in Anbetracht seines Alters zur Neoblasenfunktion wissen?

Die Kontinenzraten bei älteren PatientInnen sind insgesamt niedriger.

Die kognitive Fitness spielt für die Neoblasenentleerung keine Rolle.

Eine nervschonende Operationstechnik hat keinen Einfluss auf die Kontinenz.

Die Lebensqualität mit Neoblase ist im Alter geringer als mit Ileum-Conduit.

Selbstkatheterismus spielt nach operativ korrekter Neoblasenanlage keine Rolle.

Eine Patientin mit muskelinvasivem Blasentumor ist sich nach ausführlicher Aufklärung über die gängigen Harnableitungsarten unsicher in ihrer Entscheidung. Sie möchte von Ihnen wissen, mit welcher Harnableitung PatientInnen am glücklichsten sind?

PatientInnen mit kontinenter Harnableitung sind aufgrund des positiveren Körperbilds glücklicher als PatientInnen mit inkontinenter Harnableitung.

Die Datenlage zur Lebensqualität ist insuffizient, um einer Harnableitung klar den Vorzug zu geben.

PatientInnen mit Ureterokutaneostomie und Harnleiterschienendauerversorgung sind aufgrund der regelmäßigen Wechseltermine unglücklicher als PatientInnen mit anderen Harnableitungen.

PatientInnen mit Ileum-Conduit sind aufgrund der simpleren Handhabung der Harnentleerung glücklicher als PatientInnen mit Neoblase oder katheterisierbarem Pouch.

Unabhängig von der präoperativen Beratung sind PatientInnen mit Neoblase glücklicher als PatientInnen mit Ileum-Conduit.

Eine 84-jährige Patientin aus Ihrer Sprechstunde soll aufgrund eines muskelinvasiven Blasentumors eine Zystektomie mit einem Ileum-Conduit erhalten. Was können Sie ihr zu dieser Art der Harnableitung sagen?

Nach der Ureterokutaneostomie ist sie die zweithäufigste Technik bei PatientInnen über 80 Jahre.

Der Eingriff ist im Hinblick auf die Komplexität der Neoblase gleichzusetzten, wird aber aufgrund höherer harnableitungsspezifischer Morbidität ab dem 80. Lebensjahr bevorzugt.

Die Operationsdauer beim Ileum-Conduit ist kürzer als die bei einer Neoblase und einer Ureterokutaneostomie.

Ein Problem des Ileum-Conduits ist die lebenslang notwendige Versorgung mit Harnleiterschienen.

Das Ileum-Conduit hat auch bei PatientInnen über 80 Jahren ein bekanntes und akzeptables Risikoprofil bei guter Funktionalität.

Welche klinischen Charakteristika stellen absolute Kontraindikationen für die Anlage einer Neoblase dar?

Z. n. pelviner Radiotherapie

Schwere Niereninsuffizienz

Harnröhrenstriktur

Stressinkontinenz

Darmerkrankungen

## References

[1] IARC (2024) Estimated number of new cases in 2022, worldwide, both sexes, all ages. 2020. Access Date March

[2] Robert Koch Insitut. Krebs in Deutschland 2019/2020.; 2023.

[3] Compérat E, Larré S, Roupret M et al (2015) Clinicopathological characteristics of urothelial bladder cancer in patients less than 40 years old. virchows Arch 466(5):589–594. 10.1007/s00428-015-1739-2 25697540 10.1007/s00428-015-1739-2

[4] Witjes AJ, Bruins MH, Carrión A et al (2024) European Association of Urology Guidelines on Muscle-invasive and Metastatic Bladder Cancer: Summary of the 2023 Guidelines. Eur Urol 85(1):17–31. 10.1016/J.EURURO.2023.08.016 37858453 10.1016/J.EURURO.2023.08.016

[5] Williams SB, Huo J, Chamie K, et al. Underutilization of Radical Cystectomy Among Patients Diagnosed with Clinical Stage T2 Muscle-invasive Bladder Cancer. Eur Urol Focus. 2017;3(2–3):258–264. 10.1016/J.EUF.2016.04.008 10.1016/j.euf.2016.04.008 28753760

[6] Chamie K, Hu B, DeVere White RW, Ellison LM (2008) Cystectomy in the elderly: does the survival benefit in younger patients translate to the octogenarians? bju Int 102(3):284–290. 10.1111/J.1464-410X.2008.07636.X 18410437 10.1111/J.1464-410X.2008.07636.X

[7] Skinner EC, Stein JP, Skinner DG (2007) Surgical benchmarks for the treatment of invasive bladder cancer. Urol Oncol 25(1):66–71. 10.1016/J.UROLONC.2006.05.010 17208142 10.1016/J.UROLONC.2006.05.010

[8] Aziz A, May M, Burger M et al (2014) Prediction of 90-day mortality after radical cystectomy for bladder cancer in a prospective European multicenter cohort. Eur Urol 66(1):156–163. 10.1016/J.EURURO.2013.12.018 24388438 10.1016/J.EURURO.2013.12.018

[9] Demaegd L, Albersen M, Muilwijk T et al (2020) Comparison of postoperative complications of ileal conduits versus orthotopic neobladders. Transl Androl Urol 9(6):2541–2554. 10.21037/TAU-20-713 33457228 10.21037/TAU-20-713 PMC7807350

[10] Zhu W, Wu L, Xie W et al (2023) Comparison of morbidity and mortality after radical cystectomy between individuals older and younger than 80 years: a systematic review and meta-analysis. Int Urol Nephrol. 10.1007/S11255-023-03897-3 38095810 10.1007/S11255-023-03897-3

[11] Kontis V, Bennett JE, Mathers CD, Li G, Foreman K, Ezzati M. Future life expectancy in 35 industrialised countries: projections with a Bayesian model ensemble. Lancet (London, England). 2017;389(10076):1323–1335. 10.1016/S0140-6736(16)32381-9 10.1016/S0140-6736(16)32381-9 PMC5387671 28236464

[12] Clark PE, Stein JP, Groshen SG, et al. Radical cystectomy in the elderly: comparison of clincal outcomes between younger and older patients. Cancer. 2005;104(1):36–43. 10.1002/CNCR.21126 10.1002/cncr.21126 15912515

[13] Deutsche Gesellschaft für Geriatrie DGG. https://www.dggeriatrie.de/

[14] Fried LP, Tangen CM, Walston J et al (2001) Frailty in older adults: evidence for a phenotype. J Gerontol A Biol Sci Med Sci. 10.1093/GERONA/56.3.M146 11253156 10.1093/GERONA/56.3.M146

[15] Shaw JF, Budiansky D, Sharif F, McIsaac DI (2022) The Association of Frailty with Outcomes after Cancer Surgery: A Systematic Review and Metaanalysis. ann Surg Oncol 29(8):4690–4704. 10.1245/S10434-021-11321-2 35072860 10.1245/S10434-021-11321-2

[16] Williams SB, Kamat AM, Chamie K, et al. Systematic Review of Comorbidity and Competing-risks Assessments for Bladder Cancer Patients. Eur Urol Oncol. 2018;1(2):91–100. 10.1016/J.EUO.2018.03.005 10.1016/j.euo.2018.03.005 PMC6190914 30345422

[17] Froehner M, Brausi MA, Herr HW, Muto G, Studer UE (2009) Complications following radical cystectomy for bladder cancer in the elderly. Eur Urol 56(3):443–454. 10.1016/J.EURURO.2009.05.008 19481861 10.1016/J.EURURO.2009.05.008

[18] Wuethrich PY, Vidal A, Burkhard FC. There is a place for radical cystectomy and urinary diversion, including orthotopic bladder substitution, in patients aged 75 and older: Results of a retrospective observational analysis from a high-volume center. Urol Oncol. 2016;34(2):58.e19–58.e27. 10.1016/J.UROLONC.2015.08.011 10.1016/j.urolonc.2015.08.011 26420022

[19] Sogni F, Brausi M, Frea B et al (2008) Morbidity and quality of life in elderly patients receiving ileal conduit or orthotopic neobladder after radical cystectomy for invasive bladder cancer. Urology 71(5):919–923. 10.1016/J.UROLOGY.2007.11.125 18355900 10.1016/J.UROLOGY.2007.11.125

[20] Mortezavi A, Crippa A, Edeling S et al (2021) Morbidity and mortality after robot-assisted radical cystectomy with intracorporeal urinary diversion in octogenarians: results from the European Association of Urology Robotic Urology Section Scientific Working Group. bju Int 127(5):585–595. 10.1111/BJU.15274 33058469 10.1111/BJU.15274 PMC8246851

[21] Donat SM, Siegrist T, Cronin A, Savage C, Milowsky MI, Herr HW (2010) Radical cystectomy in octogenarians—does morbidity outweigh the potential survival benefits? j Urol 183(6):2171–2177. 10.1016/J.JURO.2010.02.015 20399461 10.1016/J.JURO.2010.02.015

[22] Huang S, Chen H, Li T, Pu X, Liu J, Bi X (2021) Comparison of survival in elderly patients treated with uretero-cutaneostomy or ileal conduit after radical cystectomy. bmc Geriatr. 10.1186/S12877-020-01861-9 33441098 10.1186/S12877-020-01861-9 PMC7807694

[23] Yamanaka K, Miyake H, Hara I, Inoue TA, Fujisawa M (2007) Significance of radical cystectomy for bladder cancer in patients over 80 years old. Int Urol Nephrol 39(1):209–214. 10.1007/S11255-006-9122-5 17082907 10.1007/S11255-006-9122-5

[24] Parekh DJ, Gilbert WB, Koch MO, Smith JA. Continent urinary reconstruction versus ileal conduit: A contemporary single-institution comparison of perioperative morbidity and mortality. Urology. 2000;55(6):852–855. 10.1016/S0090-4295(99)00619-6 10.1016/s0090-4295(99)00619-6 10840090

[25] Korkes F, Fernandes E, Gushiken FA et al (2022) Bricker ileal conduit vs. Cutaneous ureterostomy after radical cystectomy for bladder cancer: a systematic review. Int Braz J Urol 48(1):18–30. 10.1590/S1677-5538.IBJU.2020.0892 33861058 10.1590/S1677-5538.IBJU.2020.0892 PMC8691241

[26] Deliveliotis C, Papatsoris A, Chrisofos M, Dellis A, Liakouras C, Skolarikos A (2005) Urinary diversion in high-risk elderly patients: modified cutaneous ureterostomy or ileal conduit? Urology 66(2):299–304. 10.1016/J.UROLOGY.2005.03.031 16040096 10.1016/J.UROLOGY.2005.03.031

[27] Pycha A, Lodde M (2005) Uretero-Ureterocutaneostomy (Wrapped By Omentum). In: Hohenfellner R Fitzpatrick J Mcaninch J Eds advanced Urol Surg Third Ed 10.1111/j.1464-410X.2005.05302.x 15679796

[28] Kessler TM, Burkhard FC, Perimenis P, et al. Attempted nerve sparing surgery and age have a significant effect on urinary continence and erectile function after radical cystoprostatectomy and ileal orthotopic bladder substitution. J Urol. 2004;172(4 Pt 1):1323–1327. 10.1097/01.JU.0000138249.31644.EC 10.1097/01.ju.0000138249.31644.ec 15371833

[29] Yang LS, Shan BL, Shan LL et al (2016) A systematic review and meta-analysis of quality of life outcomes after radical cystectomy for bladder cancer. Surg Oncol 25(3):281–297. 10.1016/J.SURONC.2016.05.027 27566035 10.1016/J.SURONC.2016.05.027

[30] Clement KD, Pearce E, Gabr AH, Rai BP, Al-Ansari A, Aboumarzouk OM (2021) Perioperative outcomes and safety of robotic vs open cystectomy: a systematic review and meta-analysis of 12,640 cases. World J Urol. 10.1007/S00345-020-03385-8 32734460 10.1007/S00345-020-03385-8

[31] Su X, Wu K, Wang S et al (2020) The impact of orthotopic neobladder vs ileal conduit urinary diversion after cystectomy on the survival outcomes in patients with bladder cancer: A propensity score matched analysis. Cancer Med 9(20):7590–7600. 10.1002/CAM4.3404 32869540 10.1002/CAM4.3404 PMC7571812

[32] Check DK, Leo MC, Banegas MP et al (2020) Decision Regret Related to Urinary Diversion Choice among Patients Treated with Cystectomy. j Urol 203(1):159–163. 10.1097/JU.0000000000000512 31441673 10.1097/JU.0000000000000512

[33] Köther AK, Büdenbender B, Grüne B et al (2022) Different patients, different preferences: A multicenter assessment of patients’ personality traits and anxiety in shared decision making. Cancer Med 11(15):2999–3008. 10.1002/CAM4.4667 35322925 10.1002/CAM4.4667 PMC9359866

[34] Siddiqui KM, Izawa JI (2016) Ileal conduit: standard urinary diversion for elderly patients undergoing radical cystectomy. World J Urol 34(1):19–24. 10.1007/S00345-015-1706-1 26475274 10.1007/S00345-015-1706-1

[35] Hugen CM, Daneshmand S (2016) Orthotopic urinary diversion in the elderly. World J Urol 34(1):13–18. 10.1007/S00345-015-1696-Z 26410825 10.1007/S00345-015-1696-Z

[36] Longo N, Imbimbo C, Fusco F et al (2016) Complications and quality of life in elderly patients with several comorbidities undergoing cutaneous ureterostomy with single stoma or ileal conduit after radical cystectomy. bju Int 118(4):521–526. 10.1111/BJU.13462 26935245 10.1111/BJU.13462

[37] Mühlberg W, Platt D (1999) Age-dependent changes of the kidneys: pharmacological implications. Gerontology 45(5):243–253. 10.1159/000022097 10460985 10.1159/000022097

[38] Lee RK, Abol-Enein H, Artibani W et al (2014) Urinary diversion after radical cystectomy for bladder cancer: options, patient selection, and outcomes. bju Int 113(1):11–23. 10.1111/BJU.12121 24330062 10.1111/BJU.12121

[39] Lawrentschuk QLGN (2019) Orthotopic Neobladder Reconstruction: Patient Selection And Perspectives. Res reports. Urol 11:333–341. 10.2147/RRU.S181473 10.2147/RRU.S181473 PMC6912000 31850284

[40] Yajima S, Nakanishi Y, Yasujima R et al (2023) Rapid geriatric screening tools predict inability to manage stoma by oneself after urinary diversion: G8 and IADL-modified G8. J Geriatr Oncol. 10.1016/J.JGO.2023.101468 36870222 10.1016/J.JGO.2023.101468

